# GrapeMOTS: UAV vineyard dataset with MOTS grape bunch annotations recorded from multiple perspectives for enhanced object detection and tracking

**DOI:** 10.1016/j.dib.2024.110432

**Published:** 2024-04-16

**Authors:** Mar Ariza-Sentís, Kaiwen Wang, Zhen Cao, Sergio Vélez, João Valente

**Affiliations:** Information Technology Group, Wageningen University & Research, 6708 PB Wageningen, Netherlands

**Keywords:** Occlusion, UAV, Multiple view, Object detection and tracking, Precision viticulture, MOTS

## Abstract

Object Detection and Tracking have provided a valuable tool for many tasks, mostly time-consuming and prone-to-error jobs, including fruit counting while in the field, among others. Fruit counting can be a challenging assignment for humans due to the large quantity of fruit available, which turns it into a mentally-taxing operation. Hence, it is relevant to use technology to ease the task of farmers by implementing Object Detection and Tracking algorithms to facilitate fruit counting. However, those algorithms suffer undercounting due to occlusion, which means that the fruit is hidden behind a leaf or a branch, complicating the detection task. Consequently, gathering the datasets from multiple viewing angles is essential to boost the likelihood of recording the images and videos from the most visible point of view. Furthermore, the most critical open-source datasets do not include labels for certain fruits, such as grape bunches. This study aims to unravel the scarcity of public datasets, including labels, to train algorithms for grape bunch Detection and Tracking by considering multiple angles acquired with a UAV to overcome fruit occlusion challenges.

Specifications Table

**Table:** 

Subject	Agricultural Sciences, Agronomy and Crop Science
Specific subject area	Multi-View UAV Object Detection and Tracking in Agriculture
Type of data	Raw videos in MP4 format, and video frames and annotations in PNG format
Data Collection	Unmanned Aerial Vehicle: - DJI Phantom4 RTK (integrated sensor). Sensor characteristics: - Focal aperture range: f2.8 - f.11 - Shutter speed: 8-1/8000 s Flight details: - Flight speed: not stable, between 0.1 and 1 m/s. - Flight altitude: 3 m above ground level (AGL). Video Specifications: - Videos PathPlanning_1 and PathPlanning_3 have a frame width of 1920, a frame height of 1080, and a frame rate of 30.00 frames/second. - The remaining videos share the following characteristics: frame width of 3840, frame height of 2160, and 29.97 frames/second as frame rate. Videos NoPathPlanning_2 and NoPathPlanning_3 have the same frame width and height, but a frame rate of 30.00 frames/second. A total of 11 videos and their grape bunch annotations are provided. These include frames from both sides of the canopy of the vineyard rows, each having a length of approximately 110 meters. Furthermore, videos NoPathPlanning_2 and NoPathPlanning_3 also include vineyard trunks and pole labels.Video Composition:Eight videos (named PathPlanning_*) provide a multiple-angle view, each from a different vine plant. The other three videos (named NoPathPlanning_*) offer a frontal view of the canopy's side. These record the same plants as those with multiple perspectives, allowing for comparison.Recording details:The videos were captured between September 19 and September 20, 2023, during the harvesting period. Both days had sunny conditions and a wind speed below 0.5 m/s.Annotation Information:All the videos have been annotated using CVAT software [1], employing the Multiple Object Tracking and Segmentation (MOTS) annotation style [2].
Data source location	Institution: Wageningen University & Research City/Town/Region: Tomiño, Pontevedra, Galicia Country: Spain Latitude and longitude (and GPS coordinates) for collected samples/data: 41°57′18.5″N 8°47′41.2″W
Data accessibility	Repository name: Zenodo Data identification number: 10.5281/zenodo.10625595 [3] Direct URL to data: https://zenodo.org/records/10625595

## 1 Value of the Data

• Datasets, along with annotations, are helpful for researchers and professionals working with Computer Vision techniques to perform grape bunch detection and tracking [4].
• Datasets with multiple-perspective videos are crucial to avoid occlusion, which may lead to underestimation of the number of grape bunches in each row.
• Grape bunch tracking allows for counting the number of grape bunches on each side of a vineyard row, which is relevant to estimating yield. Additionally, when coupled with ground truth information in the annotations, phenotypic traits can be extracted [5], further contributing to yield estimation.
• The dataset is helpful for winegrowers and field technicians as it provides high-quality videos for visual inspection of bunch monitoring and disease development, eliminating the need to be physically present in the field.
• Datasets, together with annotations, address the lack of public agricultural datasets.
• This dataset can be integrated with other datasets from the same vineyard that contain key information such as the position of the plant trunks or lidar point clouds [4,6,7], enabling researchers to go further and achieve a more precise understanding of the vineyard.

## 2 Background

In agricultural research, the importance of datasets cannot be underestimated, and their applications in vineyards are particularly notable. They help in the identification and classification of diseases [8], as well as in the detailed analysis of yield factors [9]. Following the idea of [10], where they introduced the concept of different angles with a handheld camera to avoid occlusions and provided 11000+ images, this dataset offers Unmanned Aerial Vehicles (UAV) videos with grape bunch annotations recorded in a commercial vineyard under challenging conditions, such as occlusion. This endeavour aims not just at enriching the repository of data available for precision agriculture but also at overcoming specific hurdles not only for object detection within viticulture, similar to [11] where they provided instances to locate the bunches in the images but including tracking, by adding the same ID of each grape bunch along frames. By capturing footage from multiple vantage points around the vineyard rows, this dataset allows for a depth analysis, enabling algorithms to count bunches more accurately despite the frequent obstructions caused by foliage. Moreover, the significance of this dataset extends beyond its immediate utility. It serves as another piece that can be synergistically combined with other existing datasets from the same vineyard [4,6,7], which encompass a diverse range of data types, including videos, UAV orthoimages, and even LiDAR information. This diversity enhances the potential for data fusion and enables a multifaceted analysis of the vineyard ecosystem on the same dates but also across different years. Such comprehensive temporal and spatial coverage offers an unparalleled opportunity to study the dynamics of vineyard ecosystems in depth. Further, it empowers the available data lake of the vineyard to train models that are capable of generalizing under different operational conditions. This fusion of datasets opens up new avenues for research and application, allowing for a more detailed examination of bunch visibility, phenotypic trait extraction, and yield estimation under varying conditions, among other characteristics.

Therefore, in order to obtain a complete perspective of the vineyard, recording the side of the row from multiple perspectives becomes essential. Consequently, this dataset aids Object Detection and Tracking algorithms training in real vineyard conditions, ensuring accurate bunch counting.

## 3 Data Description

**Fig. 1:**
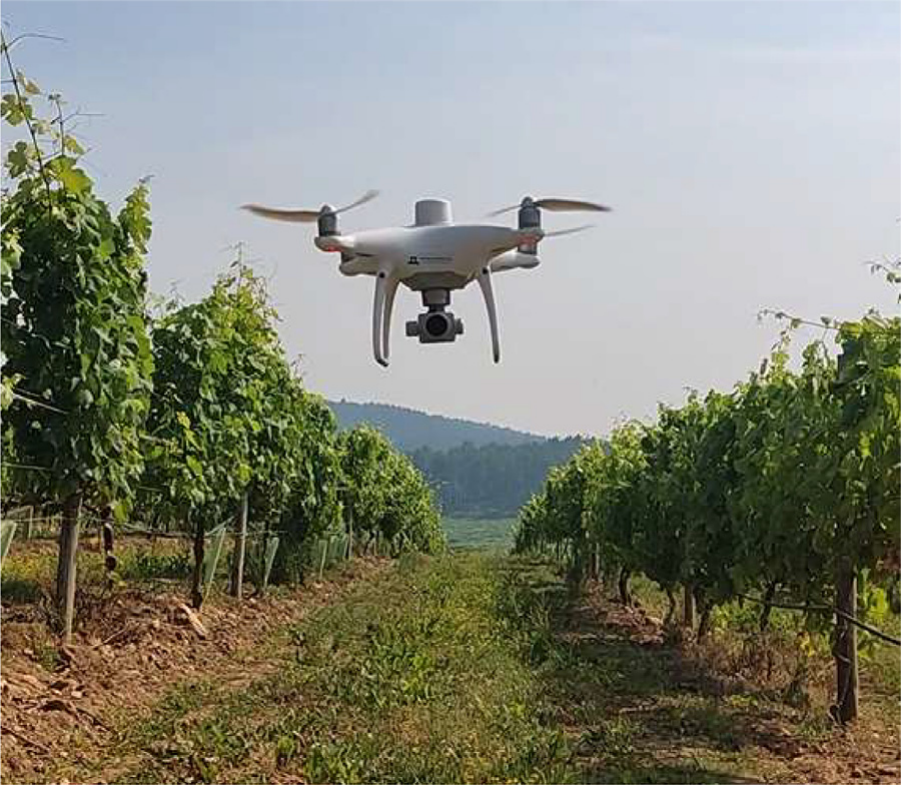
UAV taking off within the vineyard rows.

The dataset was collected during the 2023 harvesting campaign between September 19th and 20th in a 1.06-hectare commercial vineyard (*Vitis vinifera* cv. Loureiro) located in Tomiño, Spain (X: 516989.02, Y: 4644806.53; ETRS89 / UTM zone 29N) (Fig. 1). The plants, managed in a vertical trellis system, were planted in 1990 with an NE-SW orientation. The distance between rows and plants is 3 × 2.5 meters, respectively, and no leaf removal was performed, resulting in a dataset marked by leaf occlusion.

**Fig. 2:**
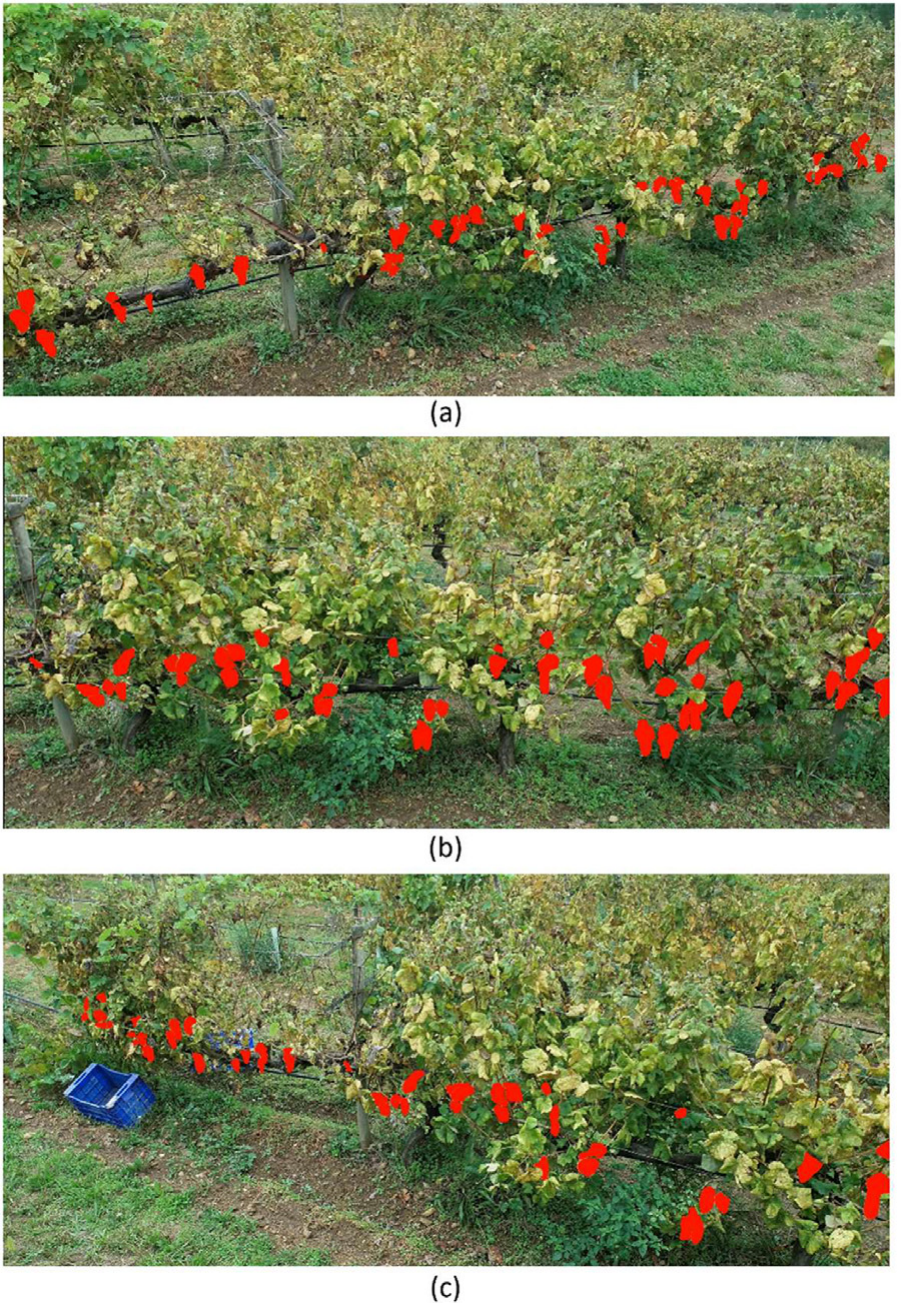
Vineyard row acquired from multiple viewing points. The red masks represent the grape bunch annotations. (a) Videos recorded from the left. (b) Vineyard row observed from a frontal point of view. (c) UAV perspective when it was recording being rotated from the right.

The dataset was collected by flying the UAV over the adjacent vineyard row, recording the side of the row of interest. Two types of videos were acquired: (1) a basic type that observed the canopy from a frontal point of view only, serving as control videos, and (2) videos following a path planning using the Ant Colony Optimization (ACO) [12,13] as optimizing algorithm with multiple-angle perspectives to address occlusion. Fig. 2 illustrates the perspectives obtained from the grape bunches when acquiring the data from multiple viewing points.

The videos with names starting with NoPathPlanning_* belong to the first category, while those starting with PathPlanning_* were recorded using ACO.

## 4 Experimental Design, Materials and Methods

The UAV platform used in this study was the DJI Phantom4 RTK (DJI Sciences and Technologies Ltd., Shenzhen, Guangdong, China), equipped with an integrated RGB sensor. The flights were conducted under a clear sky at 3 m AGL above the vineyard rows, with wind below 0.5 m/s.

### 4.1 Data annotation

A total of 11 vineyard videos were annotated using CVAT software in MOTS style for grape bunch Detection and Tracking. The MOTS annotations were labelled with per-pixel accuracy, which ensured that each grape bunch instance remained coherent throughout the video sequence. Furthermore, even shaded grape bunches were annotated to ensure proper generalization to multiple illumination scenarios. The annotation focused on exclusively labelling grape bunches, excluding the peduncle and surrounding leaves. In the videos with multiple perspectives, grape bunches appear from different viewpoints, resulting in various shapes. The same ID was maintained for grape bunches seen from different perspectives to enhance Object Tracking. In order to increase the efficiency of the annotation task and due to the similarity of adjacent frames in the video, a frame step as 2 was selected in most of the videos, except PathPlanning_1, and the three videos without Path Planning.

Table 1 summarizes the dataset, providing details on the videos, including the number of frames each video included, the number of annotated frames of each video, the frame step for each video annotation task and the size of both images and annotations. The dataset, totalling 78.8 GB excluding the original videos, includes 5958 labelled frames. Videos are available in MP4 format, while the images, along with the annotations, are provided in PNG. Moreover, the instances folder includes also a txt file, which contains the label(s) of the annotations.

**Table 1:** Description of the videos and annotations provided, along with the number of annotated frames, and the size of the zip file containing frames and instances.

Video Name	Multiple Viewing	Label	Number of annotated frames	Size (frames + instances) (GB)
PathPlanning_1	Yes	Grape bunch	927	3.8
PathPlanning_2	Yes	Grape bunch	209	3.3
PathPlanning_3	Yes	Grape bunch	400	1.7
PathPlanning_4	Yes	Grape bunch	373	6.2
PathPlanning_5	Yes	Grape bunch	270	4.3
PathPlanning_6	Yes	Grape bunch	284	4.6
PathPlanning_7	Yes	Grape bunch	224	3.2
PathPlanning_8	Yes	Grape bunch	374	5.7
NoPathPlanning_1	No	Grape bunch	898	13.6
NoPathPlanning_2	No	Grape bunch, trunk, pole	899	15.2
NoPathPlanning_3	No	Grape bunch, trunk, pole	900	16.4

## Limitations

None.

## Ethics Statement

The authors have read and followed the ethical requirements for publication in Data in Brief and confirm that the current work does not involve human subjects, animal experiments, or any data collected from social media platforms.

## Declaration of Generative AI and AI-Assisted Technologies in the Writing Process

During the preparation of this work the authors used ChatGPT in order to improve the English language and avoid orthographic errors. After using this tool/service, the authors reviewed and edited the content as needed and take full responsibility for the content of the publication.

## CRediT authorship contribution statement

**Mar Ariza-Sentís:** Investigation, Methodology, Data curation, Visualization, Writing – original draft. **Kaiwen Wang:** Investigation, Methodology, Data curation, Visualization, Writing – review & editing. **Zhen Cao:** Investigation, Methodology, Data curation, Visualization, Writing – review & editing. **Sergio Vélez:** Investigation, Methodology, Data curation, Writing – review & editing. **João Valente:** Conceptualization, Supervision, Writing – review & editing.

## Data Availability

MOTS-annotated UAV Vineyard Dataset captured using Multiple Perspectives to avoid Leaf Occlusion for Object Detection and Tracking (Original data) (Zenodo).

## References

[1] CVAT.ai Corporation, “Computer Vision Annotation Tool (CVAT).” 2022. [Online]. Available: https://github.com/opencv/cvat.

[2] Voigtlaender P. (2019). 2019 IEEE/CVF Conference on Computer Vision and Pattern Recognition (CVPR).

[3] Ariza-Sentís M., Wang K., Cao Z., Vélez S., Valente J. (2024).

[4] Ariza-Sentís M., Vélez S., Valente J. (2023). Dataset on UAV RGB videos acquired over a vineyard including bunch labels for object detection and tracking. Data Brief.

[5] Ariza-Sentís M., Baja H., Vélez S., Valente J. (2023). Object detection and tracking on UAV RGB videos for early extraction of grape phenotypic traits. Comput. Electron. Agric..

[6] Vélez S., Ariza-Sentís M., Valente J. (2023). Dataset on unmanned aerial vehicle multispectral images acquired over a vineyard affected by Botrytis cinerea in northern Spain. Data Brief.

[7] Vélez S., Ariza-Sentís M., Valente J. (2023). VineLiDAR: high-resolution UAV-LiDAR vineyard dataset acquired over two years in northern Spain. Data Brief.

[8] Alessandrini M., Calero Fuentes Rivera R., Falaschetti L., Pau D., Tomaselli V., Turchetti C. (2021). A grapevine leaves dataset for early detection and classification of esca disease in vineyards through machine learning. Data Brief.

[9] Oger B. (2023). High spatial resolution dataset of grapevine yield components at the within-field level. Data Brief.

[10] Barbole D.K., Jadhav P.M. (2023). GrapesNet: Indian RGB & RGB-D vineyard image datasets for deep learning applications. Data Brief.

[11] Santos T., de Souza L., Andreza dos S., Avila S. (2019).

[12] Dorigo M., Maniezzo V., Colorni A. (1996). Ant system: optimization by a colony of cooperating agents. IEEE Trans. Syst. Man Cybern. Part B Cybern..

[13] Stützle T., Dorigo M. (2004).

